# Synthesis and Characterization of Poly(lactic-co-glycolic) Acid Nanoparticles-Loaded Chitosan/Bioactive Glass Scaffolds as a Localized Delivery System in the Bone Defects

**DOI:** 10.1155/2014/898930

**Published:** 2014-05-11

**Authors:** K. Nazemi, F. Moztarzadeh, N. Jalali, S. Asgari, M. Mozafari

**Affiliations:** ^1^Biomaterials Group, Faculty of Biomedical Engineering (Center of Excellence), Amirkabir University of Technology, P.O. Box 15875-4413, Tehran, Iran; ^2^Computational Physiology and Biology Laboratory, Department of Computer Engineering and Computer Science, California State University, Long Beach, CA 90840, USA; ^3^Bioengineering Research Group, Nanotechnology and Advanced Materials Department, Materials and Energy Research Center (MERC), P.O. Box 14155-4777, Tehran, Iran

## Abstract

The functionality of tissue engineering scaffolds can be enhanced by localized delivery of appropriate biological macromolecules incorporated within biodegradable nanoparticles. In this research, chitosan/58S-bioactive glass (58S-BG) containing poly(lactic-co-glycolic) acid (PLGA) nanoparticles has been prepared and then characterized. The effects of further addition of 58S-BG on the structure of scaffolds have been investigated to optimize the characteristics of the scaffolds for bone tissue engineering applications. The results showed that the scaffolds had high porosity with open pores. It was also shown that the porosity decreased with increasing 58S-BG content. Furthermore, the PLGA nanoparticles were homogenously distributed within the scaffolds. According to the obtained results, the nanocomposites could be considered as highly bioactive bone tissue engineering scaffolds with the potential of localized delivery of biological macromolecules.

## 1. Introduction

In the recent years, increasing attention has been paid to biopolymers and bioactive composites for use in tissue engineering. Many of the nanocomposites are currently being used as porous scaffolds for tissue engineering applications [1]. The goal of making nanocomposites is reaching a better interaction between the bioactive inorganic phase and the organic phase, creating a tough material; therefore significant attention has been paid to the polymer/ceramic nanocomposites [2, 3].

There are many kinds of methods to provide porous scaffolds for tissue engineering, such as thermal-induced phase separation, electrospinning, gas forming foam, and freeze casting [4]. Freeze casting is a useful method because it is an environmentally friendly and economic technique [5]. Also, ceramic composites with different pore morphologies can be provided by the freeze casting method. Meanwhile, it is an effective method to avoid dried stress and shrinkage [6]. A wide variety of ceramics, such as alumina, tricalcium phosphate, titanium dioxide, hydroxyapatite, and silicon nitride, were prepared by using this method [7, 8].

Many kinds of synthetic and natural polymers have been used as scaffolds for tissue engineering such as poly(lactic acid) (PLA) and poly(glycolic acid) (PGA) and their copolymers (PLGA) for synthetic polymers that have good mechanical properties and biodegradability but have poor cell-matrix interaction [9–12]. By contrast, natural polymers such as collagen, gelatin, chitosan, and hyaluronic acid can achieve a differentiated cell phenotype and allow well cell expansion [11]. However, they have poorer mechanical properties and a faster rate of degradation [10].

Chitosan is a polymer derived from partial deacetylation of chitin. Chitosan is attractive as a suitable functional material for medical applications because it has high biodegradability, high biocompatibility, nonantigenicity, and high protein adsorption properties [13–20]. Chitosan plays an important role in the attachment, differentiation, and morphogenesis of osteoblast cells because of its structural similarities with glycosaminoglycans, an important component of bone and cartilage [19].

Bioactive glasses are a kind of bioactive ceramic materials. They have reactive surface that is used as implants in the human body to repair and replace damaged bone. They were first discovered by Hench and coworkers in 1969 [6]. Bioactive glasses have been composed mainly of SiO_2_, Na_2_O, CaO, and P_2_O_5_ [21]. They have many recognized abilities to help the growth of bone cells [22–24] and to bond strongly with hard and soft tissues. And also, bioactive glasses undergo special reactions leading to the formation of a hydroxyl carbonate apatite (HCA) layer, amorphous calcium phosphate (ACP), or crystalline hydroxyapatite (HA) phase on the surface of them, which is suitable for their strong bonding with surrounding parts [23].

Recently, Banerjee et al. [25] have reported the effect of poly(lactide-co-glycolide) (PLGA) spheres incorporation on the physical properties such as the cellular performance of the freeze-dried gelatin scaffolds. However, these effects may differ when two or more polymers used to provide porous scaffolds are mixed and these effects are largely dependent on the size of incorporated particles. Nanoparticles have many advantages over microparticles such as more homogeneous distribution of particles within the polymeric matrix during the crosslinking of scaffold fabrication and availability of much more particles for the same equivalent weight of carriers. Moreover, the lengthy diffusion times of molecules from microparticle(s) carrier matrix can be avoided when nano/submicron particles are used, which could facilitate the pulsed release of incorporated biomolecules. Another advantage with nanoparticles over microparticles is the avoidance of acidic microenvironment within particle matrix, which is a result of hydrolytic degradation of PLGA into lactic and glycolic acids [25]. Nanospheres are more attractive because of their diverse applications in the field of drug [26–30] and growth factor [26–28, 31] delivery for medical applications. It has been reported that implantation of microspheres containing growth factors resulted in improved cell phenotype and chondrogenesis [29].

In this study, we synthesized a bioactive glass named 58S using sol-gel method. Then, to mimic the mineral and organic component of natural bone, different concentrations of 58S-BG and chitosan have been mixed as nanocomposites scaffolds in which PLGA nanoparticles have been incorporated.

## 2. Materials and Methods

### 2.1. Materials

Tetraethylorthosilicate (TEOS: C_8_H_20_O_4_Si), calcium nitrate (Ca(NO_3_)_2_·4H_2_O), triethyl phosphate (TEP: C_6_H_15_O_4_P), and 0.1 M nitric acid (HNO_3_) were purchased from Merck Inc. Acetic acid (96%) and chitosan (Mw = 2.5 × 105, degree of deacetylation = 85%) were purchased from Sigma-Aldrich Co. Glutaraldehyde (GA) (C_5_H_8_O_2_) solution of 1% (w/v) was purchased from Merck Inc. Poly(DL-lactide-co-glycolide) (PLGA) 50 : 50 (RG 503 H) was purchased from Boehringer Ingelheim Pharma GmbH & Co.

### 2.2. Synthesis of 58S-BG

The 58S-BG powder (60% SiO_2_, 4% P_2_O_5_, and 36% CaO) (mol%) has been synthesized via sol-gel method. For this purpose, 108.093 mL of tetraethylorthosilicate (TEOS: C_8_H_20_O_4_Si) has been added to 39.54 mL of 0.1 M nitric acid (HNO_3_); the mixture reacted for 30 min for the acid hydrolysis of TEOS to proceed almost to completion. Then, 10.99 mL triethyl phosphate (TEP: C_6_H_15_O_4_P) and 68.66 g of calcium nitrate tetrahydrate (Ca(NO_3_)_2_ *·*4H_2_O) have been added in sequence allowing 1 h for each reagent to react completely. The solution has been cast in a cylindrical Teflon container and kept sealed for a week at room temperature to allow the hydrolysis and a polycondensation reaction to take place until the gel formed. The water has been removed and a small hole was inserted in the lid to allow the leakage of gases while heating the gel to 120°C for 2 days to remove all the water. The dried powder was heated for 24 h at 700°C for nitrate elimination and stabilization. Subsequently, the powders were milled by planetary milling (SVD15IG5-1, LG Company) at 400 rpm for 4 h.

### 2.3. Preparation of SBF Solution

The SBF solution has been prepared by dissolving reagent-grade NaCl, KCl, NaHCO_3_, MgCl_2_·6H_2_O, CaCl_2,_ and KH_2_PO_4_ into distilled water and buffered at pH = 7.25 with TRIS (trishydroxymethyl aminomethane) and 1 N HCl solution at 37°C [32]. All the reagents have been purchased from Merck Inc. Its composition is given in Table 1 and is compared with the human blood plasma.

### 2.4. Synthesis of PLGA-Loaded Chitosan/Bioactive Glass Scaffolds

The PLGA-loaded scaffolds have been prepared with different ratios of the synthesized 58S-BG as listed in Table 2. For the preparation of the PLGA-loaded scaffolds, 250 mg chitosan was dissolved in 1% acetic acid solution. Different percentages of 58S-BG have been added to the chitosan solutions and stirred for 12 h. Then, 62.5 mg of PLGA has been dissolved in 3 mL of dichloromethane and then added to the dispersing phases (0%, 0.5%, 1%, 1.5%, and 2% of 58S-BG) under moderate magnetic stirring. The PLGA nanoparticles have been formed immediately upon mixing [33]. The formed emulsions have been stirred at 1250 rpm on a magnetic stirrer plate at room temperature for 2 h to evaporate dichloromethane (DCM). The resultant solutions have been subjected to ultrasonication to reduce particle size and fine dispersions. The samples were obtained after freezing and then freeze-dried for 24 h. After soaking each scaffold into 25 mL glutaraldehyde 1% for 2 h, the samples were washed several times to remove all the unreacted glutaraldehyde molecules chains. The resultant scaffolds have been transferred into a fridge followed by freeze-drying at −80°C for 24 h.

### 2.5. Characterization

#### 2.5.1. Scanning Electron Microscope (SEM) Analysis

The morphology and microstructure of the scaffolds have been observed using a scanning electron microscope (SEM). The scaffolds have been coated with a thin layer of gold (Au) by sputtering (EMITECH K450X, England), and then the morphology of the coated samples has been observed on a SEM-Philips XL30 that operated at the acceleration voltage of 15 kV.

#### 2.5.2. Fourier Transform Infrared Spectroscopy (FTIR) Analysis

The FTIR spectra of the raw materials (chitosan, 58S-BG powder, and PLGA nanoparticles) and the prepared scaffolds have been characterized using a FTIR spectrometer (Perkin-Elmer RX1) operating at the range of 400–4000 cm^−1^. The samples have been ground and mixed thoroughly with potassium bromide at a ratio of 1 : 5 (sample : KBr).

#### 2.5.3. X-Ray Diffraction (XRD) Analysis

The apatite forming ability of the scaffolds has been analyzed by XRD (Siemens-Brucker D5000 diffractometer). This instrument works with voltage and current settings of 40 kV and 40 mA, respectively, and uses Cu-Ka radiation (1.540600 Å). For qualitative analysis, XRD diagrams were recorded in the interval 25° ≤ 2*θ* ≤ 60° at the scan speed of 2°/min with the step size 0.02° and the step time 1 s.

#### 2.5.4. Mechanical Behavior

The mechanical characteristics of the prepared scaffolds have been investigated by conducting compression strength test according to ASTM F 451-86. The cylindrical samples have been cut to an appropriate size (7 mm in diameter and 14 mm in thickness). The diameter and the thickness of the samples have been checked with an electric digital caliper. The break strength of the scaffolds has been tested by Roel-Amstel with a drawing rate of 1 mm/min.

### 2.6. Analytical Methodologies

#### 2.6.1. Density Measurement

The apparent density of the samples (*ρ*_*a*_) was measured by mercury pycnometry. A sample of weight *W*_*s*_ has been placed in a pycnometer, completely filled with mercury and weighed to obtain *W*_*s*1_. *ρ*_*a*_ was calculated according to the following equation:

(1) $$\begin{array}{c} \rho_{a}=\frac{W}{W_{1}-W_{s1}+W_{s}}\times \rho_{\text{Hg}}, \end{array}$$

where *W*_1_ is the weight of the pycnometer filled with mercury and *ρ*_Hg_ is the density of mercury (13.5 g/cm^3^).

#### 2.6.2. Swelling Index

The dried scaffolds have been accurately weighed and placed into 50 mL tubes containing 45 mL of phosphate buffered saline (PBS) solution at 37°C. The PBS solution with pH 7.4 was prepared by dissolving phosphate buffered saline tablets (Medicago Co.) in deionized water. At predetermined time intervals (1, 3, 7, and 14 days) the swollen scaffolds were wiped with soft paper tissue and weighed again. The degree of swelling for all samples at each time is calculated by the following equation:

(2) $$\begin{array}{c} \text{SI}=\left[\frac{\left(W_{st}-W_{d}\right)}{W_{d}}\right]\times 100, \end{array}$$

where *W*_*d*_ and *W*_*s*_ are the measured masses of dry and swollen scaffolds, respectively.

#### 2.6.3. Degradation in PBS Solution

The scaffolds have been immersed into the PBS solutions for degradation assessment by monitoring the weight loss. The scaffolds have been precisely weighed first and then immersed in the PBS solutions and incubated at 37°C for various periods up to 14 days without refreshing the media. After being incubated for various time durations, the scaffolds have been taken out from the PBS media, washed with deionized water repeatedly, and then immersed in deionized water to remove the traces of water-soluble inorganic ions. Subsequently, the scaffolds have been transferred into a fridge followed by freeze-drying at −80°C for 24 h and then measured. The weight loss (*W*_loss_) of the scaffolds has been calculated via the following formula:

(3) $$\begin{array}{c} W_{\text{loss}}=\left[\frac{\left(W_{\text{init}}-W_{\deg}\right)}{W_{\text{init}}}\right]\times 100, \end{array}$$

where *W*_init_ is the initial weight before degradation and *W*_deg⁡_ is the weight of the sample after degradation.

### 2.7. Statistical Analysis

All experiments were performed in five replicates. The results have been given as mean ± standard error (SE). Statistical analysis was performed by one-way ANOVA and Tukey's test with significance reported when *P* < 0.05. For investigation of group normalizing, Kolmogorov-Smirnov test has been used.

## 3. Result and Discussion

### 3.1. Morphological Observations

**Figure 1:**
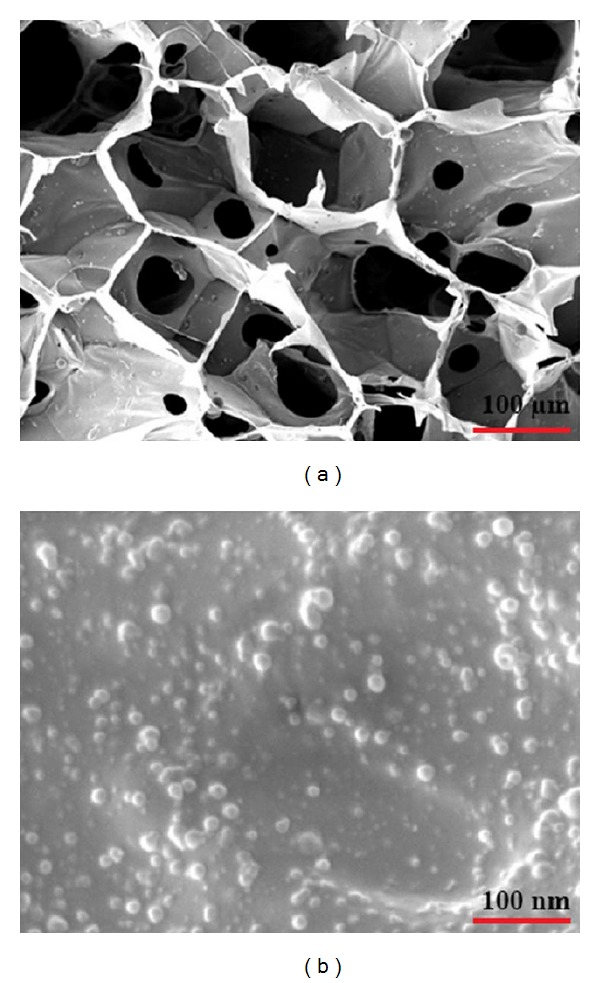
(a) Low magnification SEM micrographs of sample S3 and (b) high magnification SEM micrograph of the PLGA nanoparticles on the surface of sample S3.

The typical microstructure of the cross-section of the prepared scaffolds has been observed with SEM, shown in Figure 1. The scaffolds showed a well-developed porous structure, consisting of open interconnected pores. However, the mean pore size of the scaffolds reduced by further addition of 58S-BG. In fact, these pores are necessary for the migration and proliferation of cells in tissue engineering applications. As can be seen in Figure 1, the PLGA nanoparticles are thoroughly dispersed all over the chitosan matrix that makes it even more flexible and stronger. Under the fabrication conditions used here, the PLGA microspheres have a spherical morphology with a smooth surface and are less than 100 nm in diameter.

**Figure 2:**
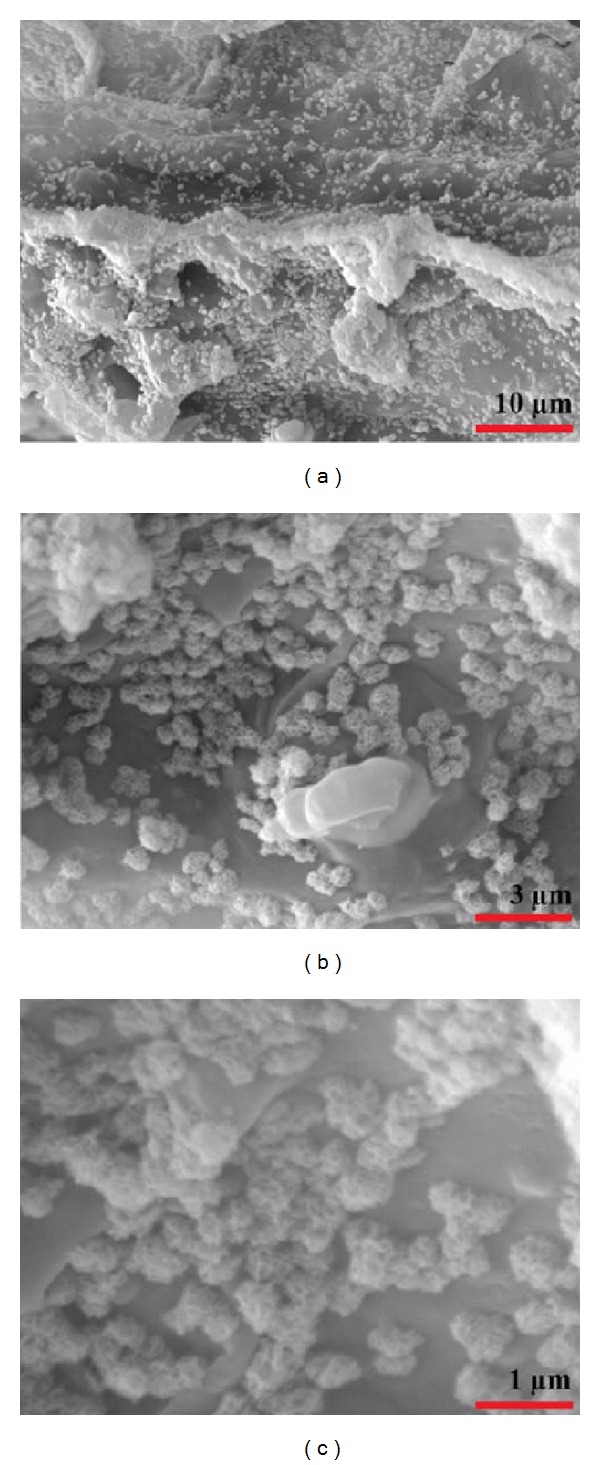
(a), (b), and (c) show higher magnification SEM micrographs of the S3 surface, respectively, after soaking in SBF solution for 7 days showing the apatite forming ability of the scaffolds.

The scaffolds have been incubated for 3 and 7 days in simulated body fluid (SBF) and their apatite forming ability was evaluated.  Figure 2 shows the SEM micrographs of the scaffold's walls after soaking in SBF solution. After 7 days, the surface has been covered with a newly formed hydroxyapatite layer as smooth spheres. Deposition of hydroxyapatite crystals demonstrated that the prepared scaffolds are highly capable of new bone formation.

### 3.2. Chemical Bondings

To reach a better understanding of the functional groups of the synthesized scaffolds, FTIR technique was employed. As a comparison, the pure components have been also analyzed. It could be seen that there was a significant difference in the whole FTIR absorbance spectra among various pure components both in the shape and in the position of the absorption peaks [34].

**Figure 3:**
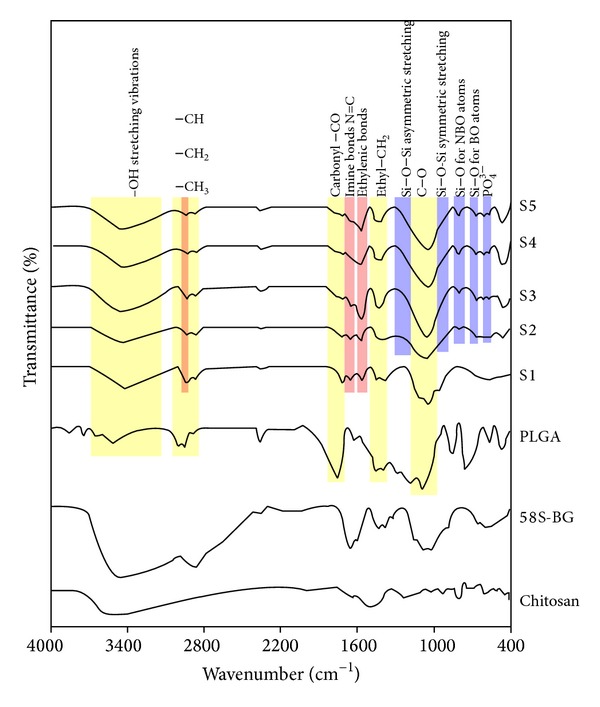
The FTIR spectra of different samples.

Figure 3 shows the FTIR spectra of scraped material surfaces. The main bands in the spectrum of all the synthesized scaffolds can be seen as follows: bands at 923 and 990 cm^−1^ (saccharide structure), bands at 1025 and 1289 cm^−1^ (deformation of amide III groups), two bands at 1563 and 1621 cm^−1^ (amide I and amide II groups), and a broad and strong overlapped band at around 3500 cm^−1^ (OH and NH stretch) [35–39].

In addition, all the synthesized scaffolds showed that the main peaks contributed to the functional groups of PLGA chemical structure, with a small shift to the lower wavenumbers, such as –CH, –CH_2_, –CH_3_ (2800–2950 cm^−1^), carbonyl –CO (1700–1760 cm^−1^), C–O (1066–1125 cm^−1^), ethyl –CH_2_ (1420 cm^−1^), and –OH stretching vibrations (3250–3500 cm^−1^) [36, 37].

The FTIR spectra of the 58S-BG containing scaffolds exhibited five significant infrared bands located at 513, 726, 812, 986, and 1131 cm^−1^ [40]. These bands, those positioned at 726, 812, 986, and 1131 cm^−1^, are related to the silicate network and, respectively, ascribed to the Si–O symmetric stretching of bridging oxygen atoms between tetrahedrons, Si–O stretching of nonbridging oxygen atoms, Si–O–Si symmetric stretching, and the Si–O–Si asymmetric stretching [1]. The band located at 513 cm^−1^ is attributed to the asymmetric vibration of PO_4_^3−^ [38, 39, 41].

The crosslinking of the scaffolds with glutaraldehyde shows the main absorption peak at 1646 cm^−1^ due to imine bonds N=C [1–3]. The shoulders at 1587 and 1713 cm^−1^ have appeared due to the ethylenic and free-aldehydic bonds, respectively. It has been also proposed that the crosslinking with glutaraldehyde can make the samples more hydrophobic as amino groups are blocked with aliphatic chains [34, 40, 42, 43].

### 3.3. XRD Analysis

**Figure 4:**
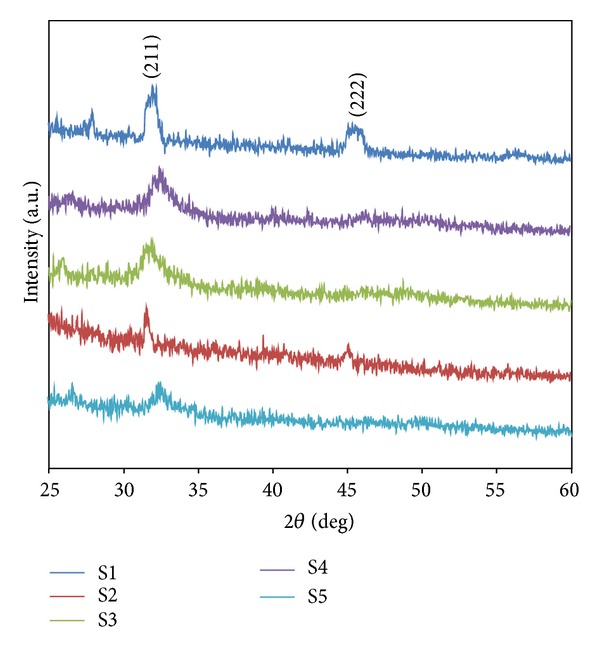
The XRD patterns of the prepared scaffolds.

To determine the bioactivity of the synthesized scaffolds, they have been subjected to in vitro solution testing using SBF solution (Figure 4). After 7 days of reaction one major peak appeared, which was located at 32° and attributed to the (211) reflection of the newly formed apatite phase and some other weak peaks appeared (JCPDS number 9-0432) [28, 33]. As can be seen, for the samples with higher amount of 58S-BG (S4), the other major peak of apatite crystals appeared at 46° attributed to the (222) reflection. The XRD diffraction peaks confirmed the formation of the apatite phase on the surface of the scaffolds. It is important to point out that the patterns showed some hydroxyapatite weak and wide reflections, indicating the formation of poorly crystalline phase of apatite.

### 3.4. Swelling Behavior

**Figure 5:**
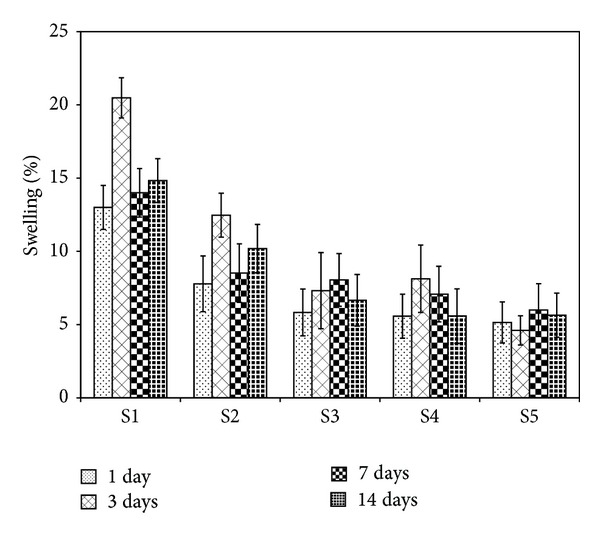
The Swelling behavior of the scaffolds in PBS solution containing lysozyme at 37°C for different time intervals.

In tissue engineering, swelling behavior is an important factor which influences the chemical and physical characteristics of the scaffolds after and prior to implantation. Herein, swelling experiments were performed after crosslinking of the synthesized scaffolds. Basically, swelling causes an increase in the pore size of the polymeric nanocomposites. In tissue engineering scaffolds, this behavior aids the supply of nutrients and oxygen to the interior regions [44]. However, scaffold's uncontrolled swelling behavior can be detrimental for tissue engineering applications. The swelling behavior of the scaffolds in PBS solution containing lysozyme at 37°C for time periods of 1, 3, 7, and 14 days is shown in Figure 5. The results suggested that by further addition of 58S-BG to the scaffolds the swelling ratio decreased. In this case it is possible to control the swelling behavior of the scaffolds. As can be seen, the swelling behavior of the scaffolds has not shown any meaningful differences in different intervals.

### 3.5. Biodegradation Behavior

**Figure 6:**
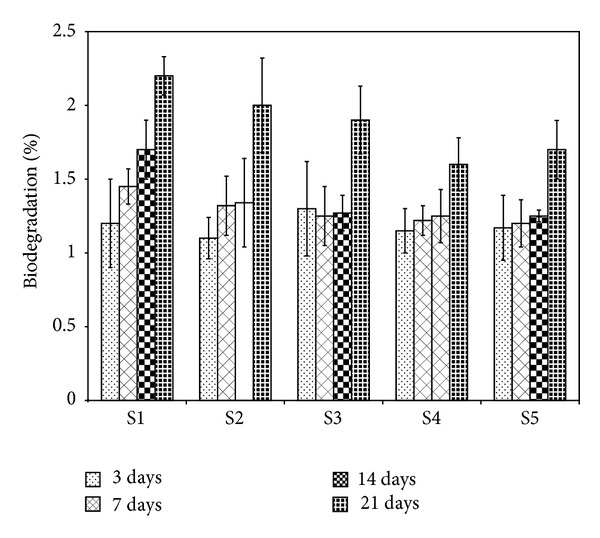
The biodegradation behavior of the scaffolds after soaking in PBS containing lysozyme at 37°C for different time intervals.

The biodegradation behavior of the scaffolds was evaluated by incubating them in PBS containing lysozyme at 37°C for time periods of 3, 7, 14, and 21 days, as shown in Figure 6. The biodegradation of the chitosan matrix can result in acidic degradation products, which may be neutralized by alkali groups leaching out from 58S-BG, thus reducing the degradation rate [45, 46]. As can be seen, the degradation test in PBS showed higher degradation behavior in longer periods especially when the samples were incubated in PBS containing lysozyme at 37°C for 21 days. In addition, by increasing the 58S-BG content inside the scaffolds, a slight decrease could be seen in the biodegradation of the scaffolds. This behavior can be related to the higher apatite formation ability of the scaffold containing higher amounts of 58S-BG content.

### 3.6. Cell Viability

**Figure 7:**
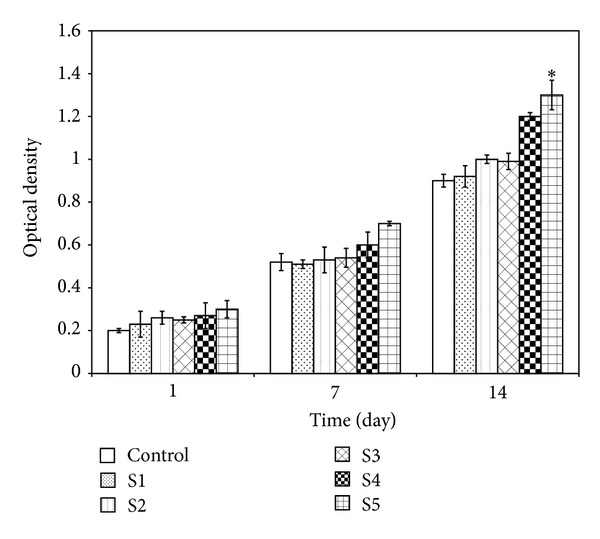
Proliferation of the osteoblastic cells when culturing them with samples (*P < 0.05).

Figure 7 shows the function of different samples in the viability of osteoblastic cells measured by MTT assay. As can be seen, there was no significant difference in formation of formazon between the control sample and the prepared scaffolds containing different amounts of 58S-BG nanoparticles after the first day (*P* < 0.05). However, the level of formazon production of cells of the scaffold samples containing higher amounts of 58S-BG was higher than the control sample after 7 and 14 days. In fact, by further addition of 58S-BG to the structure of the scaffold samples, more formazon was formed. It has been frequently reported that bioactive glasses are thought not only to have osteoconductivity but also to be responsible for osteoproduction by stimulating proliferation and differentiation of osteoprogenitor cells through a direct genetic control [47, 48]. The obtained results suggested that the osteoblastic cells could proliferate thoroughly in the presence of the prepared scaffolds.

### 3.7. Alkaline Phosphatase Activity

**Figure 8:**
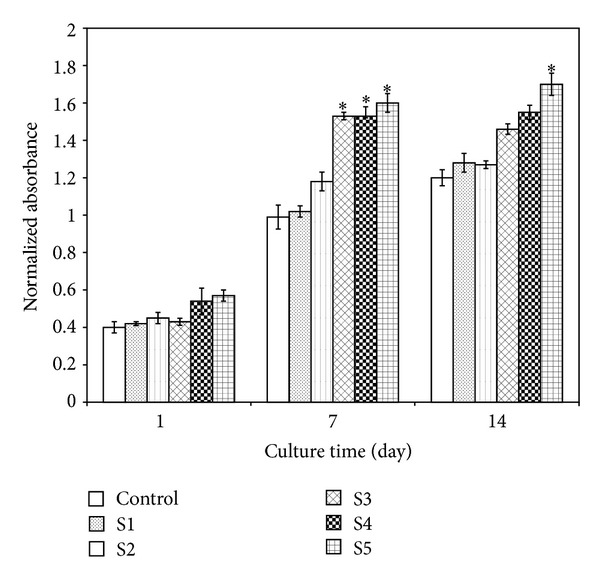
Alkaline phosphatase activity of the osteoblastic cells cultured with the samples after different time intervals.

As one of the phenotypic markers of osteoblast proliferation and differentiation is alkaline phosphatase expression, the alkaline phosphate activity of osteoblastic cells has been monitored in the presence of the control and the prepared scaffolds containing different amounts of 58S-BG nanoparticles. The osteoblastic cells in the samples were assayed for retention of their osteoblast-like phenotype and the results are shown in Figure 8. As can be seen, the level of ALP production was not statistically different for all the samples after the first day. The cell activity in the scaffold samples containing higher amounts of 58S-BG was relatively higher especially for S3, S4, and S5 samples and approximately the same level of ALP was observed for the control and S1 sample, after 7 days. The growth level of the cells was again higher in the samples containing higher amounts of 58S-BG nanoparticles (after 14 days), and in all days the maximum cell activity was observed in S5 sample. Previous studies have also suggested that the ionic dissolution products of this class of bioactive glass materials can play a vital role in the behavior of osteoblastic cells by altering the gene expression relative to osteoblast proliferation, differentiation, and bone matrix formation [49, 50]. Some other studies have proved that the osteogenetic-relative genes, such as alkaline phosphatase, bone sialoprotein, and osteocalcin, were activated by bioactive glasses [51–54]. Hattar et al. [55] have reported that the expressions of the mentioned genes could be upregulated when osteoblasts from mouse calvarias were cultured with 58S-BG particles [55].

### 3.8. Mechanical Analysis

An ideal tissue engineering scaffold should be biocompatible and highly porous with adequate mechanical properties. For this purpose the synthesized scaffolds have been tested to determine the effects of adding 58S-BG on the mechanical properties.  Table 3 gives the data obtained from mechanical compressive tests of the samples and compares them with natural cancellous bone [56, 57]. In our study, the results represented that *E* and *σ* both increased progressively by further addition of 58S-BG. It is worth mentioning that *σ* and *E* of the samples containing higher amounts of 58S-BG have been in the range of cancellous bone.

## 4. Conclusion

In conclusion, the experiments provide information to support the fabrication of the PLGA nanoparticles-loaded scaffolds in bone tissue engineering. Biomineralization studies showed the formation of apatite phase on the surface of the scaffolds ascertaining the bioactivity of the scaffolds. The scaffolds were highly porous and the elastic modulus of the scaffolds was comparative to the natural cancellous bone and also by increasing weight percentage of 58S-BG the mechanical strength increased. It is found that the swelling behavior of the scaffolds has been reduced when 58S-BG content increased. The results obtained from the degradation test in PBS showed higher degradation behavior in longer periods, and increasing the 58S-BG content inside the scaffolds could decrease the degradation of the scaffolds in some cases.

## Figures and Tables

**Table 1:** Ion concentrations of SBF and human blood plasma.

Ion	Plasma (mmol/L)	SBF (mmol/L)
Na^+^	142.0	142.0
K^+^	5.0	5.0
Mg^+2^	1.5	1.5
Ca^+2^	2.5	2.5
Cl^−^	103.0	147.8
HCO_3_^−^	27	4.2
HPO_4_^−2^	1.0	1.0
SO_4_^−2^	0.5	0.5

**Table 2:** The component of the prepared scaffolds.

Samples	Chitosan	58S-BG	PLGA
S1	1%	0%	0.25%
S2	1%	0.5%	0.25%
S3	1%	1%	0.25%
S4	1%	1.5%	0.25%
S5	1%	2%	0.25%

**Table 3:** The mechanical properties of the scaffolds from compression tests compared with cancellous bone.

Sample	*E* (MPa)	*σ*_yield_ (MPa)	*ρ* (g/cm^3^)	*E*/*ρ*	*σ*/*ρ*
Cancellous bone	**20–500**	**2–12**	**0.14–1.2**	**500**	**4–12**
S1	12.5 ± 1.2	0.8 ± 0.26	0.107 ± 0.005	116.82 ± 6.2	7.47 ± 0.12
S2	15.4 ± 2.1	1.1 ± 0.48	0.145 ± 0.005	106.20 ± 7.4	7.56 ± 0.58
S3	19.3 ± 3.9	1.5 ± 0.65	0.195 ± 0.001	98.97 ± 3.8	7.69 ± 0.32
S4	21.5 ± 1.7	2.1 ± 0.28	0.249 ± 0.014	86.34 ± 8	8.43 ± 0.17
S5	24.1 ± 1.6	2.6 ± 0.37	0.286 ± 0.008	84.26 ± 5.6	9.09 ± 0.49
